# Care Seeking for Neonatal Illness in Low- and Middle-Income Countries: A Systematic Review

**DOI:** 10.1371/journal.pmed.1001183

**Published:** 2012-03-06

**Authors:** Hadley K. Herbert, Anne CC Lee, Aruna Chandran, Igor Rudan, Abdullah H. Baqui

**Affiliations:** 1 Johns Hopkins Bloomberg School of Public Health, Department of International Health, Baltimore, Maryland, United States of America; 2 Virginia Commonwealth University Medical Center, Department of Surgery, Richmond, Virginia, United States of America; 3 Brigham and Women's Hospital, Department of Newborn Medicine, Boston, Massachusetts, United States of America; 4 Centre for Population Health Sciences and Global Health Academy, The University of Edinburgh Medical School, Edinburgh, Scotland, United Kingdom; The University of Adelaide, Australia

## Abstract

**Background:**

Despite recent achievements to reduce child mortality, neonatal deaths continue to remain high, accounting for 41% of all deaths in children under five years of age worldwide, of which over 90% occur in low- and middle-income countries (LMICs). Infections are a leading cause of death and limitations in care seeking for ill neonates contribute to high mortality rates. As estimates for care-seeking behaviors in LMICs have not been studied, this review describes care seeking for neonatal illnesses in LMICs, with particular attention to type of care sought.

**Methods and Findings:**

We conducted a systematic literature review of studies that reported the proportion of caregivers that sought care for ill or suspected ill neonates in LMICs. The initial search yielded 784 studies, of which 22 studies described relevant data from community household surveys, facility-based surveys, and intervention trials. The majority of studies were from South Asia (*n* = 17/22), set in rural areas (*n* = 17/22), and published within the last 4 years (*n* = 18/22). Of the 9,098 neonates who were ill or suspected to be ill, 4,320 caregivers sought some type of care, including care from a health facility (*n* = 370) or provider (*n* = 1,813). Care seeking ranged between 10% and 100% among caregivers with a median of 59%. Care seeking from a health care provider yielded a similar range and median, while care seeking at a health care facility ranged between 1% and 100%, with a median of 20%. Care-seeking estimates were limited by the few studies conducted in urban settings and regions other than South Asia. There was a lack of consistency regarding illness, care-seeking, and care provider definitions.

**Conclusions:**

There is a paucity of data regarding newborn care-seeking behaviors; in South Asia, care seeking is low for newborn illness, especially in terms of care sought from health care facilities and medically trained providers. There is a need for representative data to describe care-seeking patterns in different geographic regions and better understand mechanisms to enhance care seeking during this vulnerable time period.

*Please see later in the article for the Editors' Summary*

## Abstract 2

Hadley Herbert and colleagues systematically review newborn care-seeking behaviors by caregivers in low- and middle-income countries.

## Editors' Summary

**Background:**

Worldwide around 3.3 million babies die within their first month of life every year. While the global neonatal mortality rate has declined by 28% between 1990 and 2009 (from 33.2 deaths per 1,000 livebirths to 23.9), the proportion of under-five child deaths that are now in the neonatal period has increased in all regions of the world and currently stands at 41%. Of these deaths, over 90% occur in low- and middle-income countries (LMICs), making the risk of death in the neonatal period in LMICs more than six times higher than in high-income countries. In LMIC settings most babies are born at home so inappropriate and delayed care seeking can contribute substantially to neonatal mortality. Infection causes over a quarter of all deaths in neonates, but in LMICs diagnosis is often based on nonspecific clinical signs, which may delay the provision of care.

**Why Was This Study Done?:**

In order to improve neonatal survival in LMICs, health care facilities and providers must not only be available and accessible but a baby's caregiver, often a parent or other family member, must also recognize that the baby is ill and seek help. To address this problem with effective strategies, an understanding is needed of the patterns of care-seeking behavior by babies' caregivers in seeking help from health-care facilities or providers. In this study, the researchers explored the extent and nature of care-seeking behaviors by the caregivers of ill babies in LMIC settings.

**What Did the Researchers Do and Find?:**

Using multiple databases, the researchers conducted a comprehensive review up until October 2011 of all relevant studies including those that had not been formally published. Using specific criteria, the researchers then identified 22 appropriate studies (out of a possible 784) and recorded the same information from each study, including the number of neonates with illness or suspected illness, the number of care providers who sought care, and where care was sought. They also assessed the quality of each included study (the majority of which were from rural areas in South Asia) on the basis of a validated method for reviewing intervention effectiveness. The researchers found that the definitions of neonatal illness and care-seeking behavior varied considerably between studies or were not defined at all. Because of these inherent study differences it was not possible to statistically combine the results from the identified studies using a technique called meta-analysis, instead the researchers reported literature estimates and described their findings narratively.

The researchers' analysis included 9,098 neonates who were identified in community-based studies as being ill or suspected of being ill and a total of 4,320 related care-seeking events: care seeking ranged between 10% and 100% among caregivers including seeking care from a health facility (370) or from a health provider (1,813). Furthermore, between 4% to 100% of caregivers sought care from a trained medical provider and 4% to 48% specified receiving care at a health care facility: caregivers typically sought help from primary health care, secondary health care, and pharmacies and some from an unqualified health provider. The researchers also identified seven community-based intervention studies that included interventions such as essential newborn care, birth preparedness, and illness recognition, where all showed an increase in care seeking following the intervention.

**What Do These Findings Mean?:**

These findings highlight the lack of a standardized and consistent approach to neonate care-seeking behaviors described in the literature. However, despite the large variations of results, care seeking for newborn illnesses in LMICs appears to be low in general and remains a key challenge to improving neonatal mortality. Global research efforts to define, understand, and address care seeking, may help to reduce the global burden of neonatal mortality. However, to achieve sustainable improvements in neonatal survival, changes are needed to both increase the demand for newborn care and strengthen health care systems to improve access to, and quality of, care. This review also shows that there is a role for interventions within the community to encourage appropriate and timely care seeking. Finally, by addressing the inconsistencies and establishing standardized terms to identify barriers to care, future studies may be able to better generalize the factors and delays that influence neonatal care seeking.

**Additional Information:**

Please access these Web sites via the online version of this summary at http://dx.doi.org/10.1371/journal.pmed.1001183.

- A recent PLoS Medicine study has the latest figures on neonatal mortality worldwide
- UNICEF provides information about progress toward United Nations Millennium Development Goal 4
- UNICEF also has information about neonatal mortality
- The United Nations Population Fund has information on home births

## Introduction

As the field of public health continues to strive to reach the fourth United Nations Millennium Development Goal (MDG) to reduce child mortality, a systematic analysis of the progress made indicates that there has been limited advancement to reduce the rate of neonatal mortality [1]. While the mortality of children under five years of age has decreased worldwide from 10.8 million deaths per year in 2000 to 8.8 million deaths in 2008, neonatal deaths decreased only from 3.9 million to 3.6 million during that time. This number represents an increased proportion of neonatal to under-five child deaths from 36% in 2000 to 41% in 2008 [1]. Of these deaths, over 90% occur in low- and middle-income countries (LMICs), making the risk of death in the neonatal period in LMICs more than six times the risk in high-income countries [2].

Of the several contributors to neonatal mortality, infections are the single largest cause of death, responsible for an estimated 25% of all neonatal deaths [3]. Neonatal sepsis has traditionally been defined as bacteremia with hemodynamic compromise and systemic signs of infection. However, in LMICs, continuous vital sign monitoring, blood cultures, and other confirmatory laboratory tests may not be available and thus the diagnosis of neonatal sepsis is often based on clinical signs. These signs are frequently nonspecific and may include lethargy or irritability, poor feeding, vomiting, respiratory distress, apnea, fever, and hypothermia [4]. Inappropriate and delayed care seeking can contribute substantially to the resulting neonatal mortality.

Effective strategies to improve survival from neonatal infections in LMICs require a clear understanding of neonatal care-seeking behaviors and patterns by caregivers. Care-seeking behaviors are often described in the literature by either the type of health care facility or provider to which the neonate presented. Additionally, to deliver successful neonatal health care interventions, health care facilities and providers must not only be available and accessible, but illness must first be recognized and care desired by the neonate's caregiver, often a parent or other family member. As such, understanding care-seeking practices becomes essential for health interventions to have a positive impact.

The overall aim of this literature review is to describe the proportion of caregivers who seek medical care once they recognize their neonate is ill or is suspected to be ill in LMICs. This review provides data to model the potential impact of point of care testing for neonatal infections, given the state of current care-seeking patterns in LMICs.

## Methods

A systematic literature search was conducted between September and October, 2011 of the following databases: PubMed/Medline [5], Embase [6], the Cochrane Library [7], the Global Health Library [8], African Index Medicus [9], African Trials Register [10], Africa-Wide Information [11], and Literature on the Health Sciences in Latin America and the Caribbean (LILACS) [12]. “Grey” literature sources were searched to identify program and unpublished reports, including search engines Eldis [13], Data Online for Population, Health and Nutrition (DOLPHN) [14], Reproductive Health Gateway [15], donor websites (Basic Support for Institutionalizing Child Survival [BASICS] [16] and Saving Newborn Lives [17]), Demographic and Health Surveys (DHS) [18], and Service Provision Assessments (SPA) [19]. Search terms were generated using key words and mesh headings for care seeking, neonates, and LMICs. See Texts S1 and S2 for the study's PRISMA checklist and protocol. A complete list of search terms, formatted for PubMed, is provided in the study protocol. The review is registered with PROSPERO (registration number CRD42011001654) [20].

Database searches were not limited by date. Language restrictions were limited to those articles published in the Latin alphabet and articles that required translation to English were translated by the authors. Additional articles were identified from the bibliographies of the articles reviewed through snowball sampling [21]. All citations were imported into an electronic database (Refworks, Proquest). Two reviewers independently assessed the studies identified during the screening search using inclusion/exclusion criteria.

### Inclusion/Exclusion Criteria

Studies were included if they met the following inclusion criteria: (1) the publication was a study of original work; (2) the study was conducted in a LMIC, using the World Health Organization's (WHO) definition of LMICs [22]; (3) the study quantitatively specified care seeking for neonates, defined as an infant less than or equal to 28 d of age. If the study did not specify the exclusion of neonates from the study, corresponding authors were contacted for further clarification of their study sample; (4) the study specified that care was sought for an illness or a suspected illness, opposed to non-illness-related complications, such as prematurity, intrapartum complications, tetanus, or congenital abnormalities.

Articles were excluded if they did not specifically identify neonates, specify care-seeking behaviors, or occur in a LMIC. In terms of study design, four types of publications and studies were excluded: (1) review articles or editorials, because they did not provide primary data; (2) intervention studies that offered care in the home setting and explored themes of care acceptance, because acceptance of care in a trial scenario was considered to be beyond the scope of this care-seeking review; (3) qualitative studies that described determinates of care seeking and not care-seeking events, which was also considered beyond the scope of this review; and (4) publications that described a duplicate study population. If this occurred, the subsidiary publication was excluded.

### Definitions

Neonatal illness was defined according to the definitions provided by the included studies. Care seeking was defined as any care sought for a neonate that was perceived by the caregiver to be ill, sick, or septic. A caregiver was defined as the individual who sought care for the ill neonate, as identified by the study, and was often a family member, such as a mother or grandmother. Type of care sought was categorized according to facility type, health care provider type, home care, or no care. Facility type was categorized as either primary or secondary health centers or pharmacies, as identified by each study. Primary health centers included public and private clinics, health centers, and out-patient care; secondary health centers included public and private hospitals and health facilities with in-patient care. Health care providers included medically trained providers and unqualified providers. Medically trained providers included government providers (GPs), who were qualified medical practitioners employed at government hospitals, nongovernmental consultants (NGCs), who were health care providers that worked through privately owned clinics and hospitals, and paramedics [23]. Unqualified providers included unqualified village doctors, traditional healers, spiritual healers, unqualified allopaths, homeopaths, and nongovernmental dispensers (NGDs), defined as self-employed health care providers who could dispense medicines without prescriptions [23]. Home care included any care provided by family members or neighbors in a home setting.

### Data Extraction and Assessment of Study Quality

From each included study the following were abstracted: study location, design, setting, the number of subjects included in the study population, the number of neonates with an illness or suspected illness, the number of caregivers that sought care, and where care was sought. To facilitate a comparison of where care was sought, type of care was grouped in terms of facility or provider type. All care-seeking encounters were extracted from the data and presented in this review, including multiple types of care sought by one caregiver. If the study did not specify the number of neonates with an illness or a suspected illness or did not provide the number of caregivers that sought care, corresponding authors were contacted to obtain this information directly.

Study quality was assessed on the basis of a modification of methods for systematic reviews for intervention effectiveness described by the Child Health Epidemiology Reference Group (CHERG), using principles relevant for the aims of this particular review [24]. Each study was evaluated by study design, population representativeness, the quality and consistency of definitions, generalizability to the population of interest, and precision in terms of the number of reported care-seeking events. “Study design” was defined according to whether data were prospectively or retrospectively collected and potentially influenced by recall bias. “Population representativeness” described the extent to which the study sample was representative of the general population as being either population or health facility based with minimal or moderate bias. “Quality of definitions” described the extent to which a study defined neonate illness, care seeking, type of health care sought. “Consistency” was assessed across all studies to ascertain the extent to which these definitions were similar. “Generalizability” was defined according to the degree to which results could be applied to other settings and populations of interest. “Precision” was defined as the extent to which the study populations included a sufficient sample size. If a study's total study population was less than 50, the quality of the evidence was considered insufficient to generalize the effect of the outcome to the target population [24].

### Data Synthesis

Due to study heterogeneity, a meta-analysis was not possible. Therefore, we reported the literature estimates and described those data narratively. To minimize the inherent selection bias of facility-based surveys regarding their population sample and care-seeking behaviors, we did not include studies that were facility-based in our description of the total number of ill neonates, the total number of care-seeking events, and the type of care sought. Additionally, when describing data from intervention trials, including randomized control trials (RCT) and before/after interventions, we included data pertaining to neonates who were either in the control or “before” groups to minimize the interventions' effect on caregivers' initial care-seeking behaviors.

## Results

**Figure 1:**
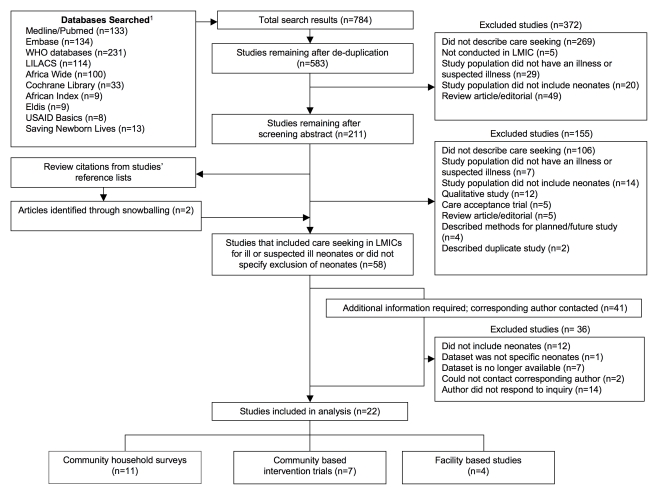
Literature flowchart of care seeking for ill or suspected ill neonates. Footnote 1: The following databases did not yield any search results: UNICEF, Reproductive Health Gateway, Dolphn, DHS, SPA, and African Trials.

Our initial search yielded 784 citations; after excluding duplicate articles and review of studies' titles and abstracts, 211 articles were selected for full-text review (Figure 1). Of these, 155 articles were excluded on the basis of exclusion criteria. Two additional studies were identified by bibliography review. Of the remaining 58 articles, 41 articles did not specify exclusion of neonates or required additional information pertaining to the number of ill neonates and/or care-seeking events; of these, the corresponding authors of 39 studies were contacted for further information (two authors could not be located). Twenty-four authors responded to our inquiry, providing the necessary information to apply our inclusion and exclusion criteria.

A total of 22 articles met our inclusion criteria, of which 11 studies were community-based surveys, seven were intervention trials, and four were facility-based surveys (Table 1). The majority of the studies occurred in the Southeast Asia Region (*n* = 17/22). The remaining five studies occurred in Africa. All studies were published after 2000 and the majority were published after 2007 (*n* = 18/22). Thirteen studies were cross-sectional surveys, of which 11 were community based and two were facility based. Five studies were cluster RCTs (c-RCT), three were “before and after” interventions, and one was a prospective follow-up study. The majority of studies were conducted in rural settings (*n* = 17/22); of the remaining studies, three occurred in urban settings, one in a peri-urban setting, and one in both urban and rural settings.

**Table 1:** Characteristics of studies that describe care seeking for ill or suspected ill neonates.

Primary Author, Year	Country	Rural/Urban	Study Design	Study Setting	Study Sample (*n*)	Neonates with Illness/Suspected Illness (n)	*n* Caregivers Who Sought Care by Type of Care Sought (%)^a^
**Community Household Surveys**							
**South Asia**							
Ahmed, 2001 [55]	Bangladesh	Rural	Cross-sectional community survey	4 rural subdistricts, Chittagong and Jessore	1511,511	740	Any care: 644 (87%); From facility: 30 (4%); From provider: 614 (82%)
Bhandari, 2002 [56]	India	Urban	Cross-sectional community survey	Urban slum, Delhi	21	9	Any care: 2 (22%); From facility: 2 (22%)
Mohan, 2008 [57]	India	Rural	Cross-sectional community survey	Rural district, Rajasthan	290	202	Any care: 63 (31%); From facility: 24 (12%); From provider: 20 (10%)
Baqui, 2008 [58] b	Bangladesh	Rural	Cross-section community survey, nested in C-RCT	Sylhet district	Baseline: 5,577	Baseline: 1,226	Baseline: Any care: 498 (41%); From provider: 498 (41%)
Dongre, 2008 [59]	India	Rural	Cross-sectional community survey	Rural district, Wardha	1160	321	Any care: 134 (42%); From facility: 56 (17%)
Dongre, 2009 [60]	India	Urban	Cross-sectional community survey	Field practice area, Wardha	72	27	Any care: 27 (100%); From provider: 27 (100%)
Willis, 2009 [61]	India	Rural	Cross-sectional community survey	Rural block, Uttar Pradesh	255	158	Any care: 120 (76%); From provider: 62 (39%)
Chowdhury, 2011 [62]	Bangladesh	Rural	Cross-sectional community survey	Rural subdistrict, Matlab	365	365	Any care: 228 (62%); From facility: 2 (1%); From provider: 226 (62%)
**Africa**							
Bazzano, 2008 [63]	Ghana	Rural	Cross-sectional community survey	3 villages and 1 town, Kintampo District	2,878	59	Any care: 36 (61%); From provider: 36 (61%)
Manji, 2009 [31]	Tanzania	Urban/rural	Cross-sectional community survey	N/A	N/A	N/A	Any care: N/A (100%)
Waiswa, 2010 [64] ^2^	Uganda	Rural	Cross-sectional health center survey	16 health facilities	64	6	Any care: 6 (100%); From facility: 6 (100%)
**Community Based Intervention Studies**							
**South Asia**							
Manandhar, 2004 [26]	Nepal	Rural	C-RCT	42 clusters, Makwanpur district	Control: 3,226	Control: 1,320	Control: Any care: 131 (10%); From facility: 131 (10%);
					Intervention: 2,899	Intervention: 919	Intervention: Any care: 219 (24%); From facility: 219 (24%)
Kumar, 2008 [28]	India	Rural	C-RCT	Household survey	Control: 988	Control: 296	Control: Any care: 254 (86%); From provider: 235 (80%)
					ENC: 1,458	ENC: 319	ENC: Any care: 257 (81%); From provider: 230 (73%)
					ENCT: 1,039	ENCT: 227	ENCT: Any care: 204 (89%); From provider: 182 (80%)
McPherson, 2008 [30]	Nepal	Rural	Before/after intervention	106 rural villages, Siraha District	N/A	N/A	Before: Any care: N/A (78%); After: Any care: N/A (85%)
Dongre, 2009 [65]	India	Rural	Before/after intervention	23 villages, Wardha	Before: 404	Before: 246	Before: Any care: 119 (48%); From facility: 119 (48%)
					After: 393	After: 147	After: Any care: 114 (78%); From facility: 114 (78%)
Azad, 2010 [27]	Bangladesh	Rural	C-RCT	Three rural districts, Bogra, Faridpur, Moulavibazar	Control: 3,227	Control: 923	Control: Any care: 244 (24%)
					Intervention: 3,162	Intervention: 866	Intervention: Any care: 195 (23%)
Darmstadt, 2010 [29]	Bangladesh	Rural	C-RCT	Rural subdistrict, Mirzapur	Control: 5,241	Control baseline: 812	Control baseline: Any care: 763 (94%); From provider: 222 (27%)
						Control endpoint: 400	Control endpoint: Any care: 384 (96%); From provider: 138 (35%)
					Intervention: 4,616	Intervention baseline: 733	Intervention baseline: Any care: 682 (93%); From provider: 225 (31%)
						Intervention endpoint: 355	Intervention endpoint: Any care: 344 (97%); From provider: 198 (56%)
Tripathy, 2010 [25]	India	Rural	C-RCT	36 clusters, Jharkhand and Orissa	Control: 8,867	Control: 2,388;	Control: Any care: 1050 (44%)
					Intervention: 9,468	Intervention: 1,739	Intervention: Any care: 940 (54%)
**Facility-Based Studies**							
**South Asia**							
Awasthi, 2006 [66]	India	Rural	Cross-sectional hospital survey	2 public hospitals, Lucknow	200	79	Any care: 46 (56%); From provider: 46 (56%)
Awasthi, 2008 [67]	India	Urban	Prospective follow-up study	Urban city, Lucknow	289	79	Any care: 64 (81%); From provider: 64 (81%)
Awasthi, 2009 [23]	India	Urban	Before/after intervention	Rural village, Sarojininagor Block	510	Before: 242	Before: Any care: 196 (81%); From provider: 111 (46%)
						After 217	After: Any care: 192 (89%)
**Africa Region**							
Ogunlesi, 2011 [68]	Nigeria	Urban	Cross-sectional health center survey	1 public hospital, Sagamu	182	182	Any care: 144 (79%); From facility: 144 (79%)

a Percentage reported as a proportion of all neonates with an illness or suspected illness reported by each study.

b Data obtained via correspondence with corresponding author.

Abbreviations: C-RCT, cluster randomized controlled trial; CHW, community health worker; ENC, essential newborn care; ENCT, essential newborn care plus thermostat; N/A, not available.

### Percentage of Sick Newborns Seeking Care Outside of the Home

The included studies identified a total of 9,680 neonates who were ill or suspected of being ill. After controlling for facility-based studies, 9,098 ill neonates were identified in community-based studies as being ill or suspected of being ill, of which there were a total of 4,320 care-seeking events. Among the non-facility-based studies, 370 care-seeking events occurred at a health care facility and 1,813 occurred with a provider. Care seeking by caregivers for any care ranged between 10% and 100%, with a median of 59%. Care seeking from a health care provider yielded the same range and median, while care seeking at a healthcare facility ranged between 1% and 100%, with a median of 20%.

All community-based intervention studies occurred in the Southeast Asia Region and used interventions such as women's groups and action learning cycle (*n* = 3/7) [25]–[27], essential newborn care (ENC; *n* = 2/7) [28],[29], behavior change and illness recognition (*n* = 1/7), birth preparedness (BPP; *n* = 1/7) [30]. All studies showed an increase in care seeking following the given intervention.

### Quality Assessment

While all studies described caregivers that sought some form of care, eight studies described caregivers who sought care from a facility and 12 describe care sought from a provider. Table 2 presents a summary of data for these care-seeking categories, while Table 3 describes the quality of each individual study. Of the 22 studies, more than half of the studies were retrospective (*n* = 14), had recall periods of less than 2 mo (*n* = 13), and were population based with minimal bias (*n* = 14). Four studies did not define neonatal illness and two studies did not define care seeking or the type of care sought. In general, the studies reported a wide range of definitions to define neonatal illness. Illness definitions ranged from illness diagnosed by documenting one or more clinical danger signs or complication, to specific danger signs (such as sepsis or pneumonia; the presence of a cough, fever, or diarrhea; or the presence of jaundice), to not being defined. Definitions of care seeking ranged from any care, to care sought outside the home or from a specific health care facility or provider, to not being defined. While two studies did not state their sample size, only one study had a sample size of less than 50 neonates. Because of the inherent selection bias of facility-based surveys regarding their population sample and their care-seeking behaviors, facility-based studies were excluded from further descriptions regarding type of care sought.

**Table 2:** Quality of data regarding care seeking for ill or suspected ill neonates: summary of included studies.

*n* Studies	Study Design		Population Representativeness	Definitions of Illness, Care Seeking, and Type of Health Care		Generalizability to Population of Interest	Precision: Study Sample with >50 Neonates
	Prospective Versus Retrospective	Recall Period		Quality of Definitions	Consistency		
*Studies that describe caregivers that sought any care*							
**22** [23],[25]–[31],[55]–[68]	Prospective (*n* = 9); Retrospective (*n* = 14)	≤2 mo (*n* = 13); ≤1 y (*n* = 6); >1 y (*n* = 1); N/A (*n* = 2)	Population based with minimal bias (*n* = 14); Population based with moderate bias (*n* = 3); Facility-based with minimal bias (*n* = 3); Facility-based with moderate bias (*n* = 1); N/A (*n* = 1)	Defined illness (*n* = 18); Defined care seeking (*n* = 20); Defined type of health care (*n* = 20)	Wide range of inconsistency of definitions	Rural (*n* = 17); Urban (*n* = 3); Peri-urban (*n* = 1); Urban/Rural (*n* = 1)	>50 neonates (*n* = 19); ≤50 neonates (*n* = 1); N/A (*n* = 2)
*Studies that describe caregivers that sought care at a health care facility*							
**8** [26],[55],[57],[59],[62],[64],[65],[68]	Prospective (*n* = 3) Retrospective (*n* = 5)	≤2 mo (*n* = 5); ≤1 y (*n* = 2); N/A (*n* = 1)	Population based with minimal bias (*n* = 5); Population based with moderate bias (*n* = 2); Facility-based with moderate bias (*n* = 1)	Defined illness (*n* = 7); Defined care seeking (*n* = 8); Defined type of health care (*n* = 8)	Wide range of inconsistency of definitions	Rural (*n* = 7); Urban (*n* = 1)	>50 neonates (*n* = 8)
*Studies that describe caregivers sought care from a health care provider*							
**12** [23],[28],[29],[55],[57],[58],[60]–[63],[66],[67]	Prospective (*n* = 3); Retrospective (*n* = 9)	≤2 mo (*n* = 9); ≤1 y (*n* = 2); >1 y (*n* = 1)	Population based with minimal bias (*n* = 8); Population based with moderate bias (*n* = 1); Facility-based with minimal bias (*n* = 3)	Defined illness (*n* = 11); Defined care seeking (*n* = 12); Defined type of health care (*n* = 12)	Wide range of inconsistency of definitions	Rural (*n* = 9); Urban (*n* = 2); Peri-urban (*n* = 1)	>50 neonates (*n* = 12)

Abbreviations: N/A, not available.

**Table 3:** Quality of data regarding care seeking for ill or suspected ill neonates: all included studies.

Primary Author, Year	Study Design		Population Representativeness: Description of the Study Population	Quality of Definitions: Study Definitions of Illness, Care Seeking, and Type of Health Care	Generalizability: Rural or Urban	Precision: Study Sample Size >50 Neonates?
	Prospective or Retrospective	Recall Period				
**Community household surveys**						
**South Asia**						
Ahmed, 2001 [55]	Retrospective	2-mo recall period	**Population based with minimal bias**: Mothers identified from a sample registration system	**Illness:** a morbid condition, including difficulty breathing, fever, convulsion, diarrhea, boils, jaundice, umbilical redness/discharge, loss of weight, oral ulcer, cold, rash, or other; **Care seeking:** sought care from specific health care provider or facility; **Type of health care:** homeopath, village doctor, TH, government facility, private doctor, other	Rural	Yes
Bhandari, 2002 [56]	Retrospective	4-mo recall period	**Population based with moderate bias:** Neonatal deaths	**Illness:** pneumonia, sepsis, or meningitis; **Care seeking:** care from public hospital; **Type of health care:** public hospital	Rural	No
Mohan, 2008 [57]	Retrospective	45-d recall period	**Population based with minimal bias:** Live births in study population	**Illness:** at least one danger sign of fever, refusal to breast-feed, convulsions, lethargy, blood in stool, rapid breathing, or chest in-drawing; **Care seeking:** any care outside the home; **Type of health care:** any health care facility	Rural	Yes
Baqui, 2008 [58]	Retrospective	1-y recall period	**Population based with minimal bias:** Women had given birth within one y preceding survey	**Illness:** poor feeding, diarrhea, umbilical redness/discharge, red or discharging eyes, difficulty or fast breathing, chest in-drawing, jaundice, fever, convulsions, lack of crying, or unconsciousness; **Care seeking:** care from trained providers; **Type of health care:** trained providers (physicians, nurses, or family welfare assistants)	Rural	Yes
Dongre, 2008 [59]	Retrospective	1-y recall period	**Population based with minimal bias:** Mothers with a child <1 y of age	**Illness:** one or more IMNCI danger sign; **Care seeking:** any care; **Type of health care:** home care versus government hospital or private hospital	Rural	Yes
Dongre, 2009 [60]	Retrospective	1-y recall period	**Population based with minimal bias:** Women identified through a mapping exercise	**Illness:** not defined; **Care seeking:** care from medical doctor or TH; **Type of health care:** medical doctor or TH	Peri-urban	Yes
Willis, 2009 [61]	Retrospective	4-wk recall period	**Population based with minimal biased:** Pregnant women in study area, excluding those planning to deliver at a health care facility	**Illness:** perceived neonatal morbidities; **Care seeking:** any care; **Type of health care:** any health care resources	Rural	Yes
Chowdhury, 2011 [62]	Retrospective	2- to 6-wk recall period	**Population-based with moderate bias:** Neonatal deaths	**Illness:** fatal illness; **Care seeking:** care from health care provider; **Type of health care:** qualified provider (MBBS or paramedic), *Kabiraj*, unqualified allopath, homeopath, spiritual leader, pharmacy	Rural	Yes
**Africa**						
Bazzano, 2008 [63]	Retrospective	4-wk recall period	**Population based with minimal bias:** Women enrolled in Obaapa Vit A trial	**Illness:** not defined; **Care seeking:** any care outside the home; **Type of health care:** doctor/hospital versus TH	Rural	Yes
Manji, 2009 [31]	Retrospective	N/A	**N/A:** Sample population not stated	**Illness:** not defined; **Care seeking:** not defined; **Type of health care:** not defined	Urban/rural	N/A
Waiswa, 2010 [64]	Retrospective	4- to 6-wk recall period	**Population-based with moderate bias:** Neonataldeaths	**Illness:** sepsis or pneumonia; **Care seeking:** any care outside the home; **Type of health care:** health facility, drug shop, spiritual leader, friend/relative	Rural	Yes
**Community based intervention studies**						
**South Asia**						
Manandhar, 2004 [26]	Prospective	N/A	**Population based with minimal bias:** Married women 15–49 y with potential to become pregnant	**Illness:** any of the three illnesses (cough, fever, diarrhea); **Care seeking:** health facility; **Type of health care:** health facility	Rural	Yes
Kumar, 2008 [28]	Prospective	3-y recall period	**Population based with minimal bias:** Live births in study population	**Illness:** danger sign recognition; **Care seeking:** any care; **Type of health care:** nurse, doctor, unqualified medical practitioner, TH, other (family members)	Rural	Yes
McPherson, 2006 [30]	Prospective	1-y recall period	**Population based with minimal bias:** Mothers of live infants less one y of age at the time	**Illness:** not defined; **Care seeking:** not defined; **Type of health care:** not defined	Rural	N/A
Dongre, 2009 [65]	Prospective	1-y recall period	**Population based with minimal bias:** Mothers with a child less than one y	**Illness:** one or more danger sign; **Care seeking:** care from government hospital or from private hospital; **Type of health care:** government hospital or private hospital	Rural	Yes
Azad, 2010 [27]	Prospective	6 wk after delivery	**Population based with minimal bias:** Women aged 15–49 y, residing in study area, who gave birth during the study period	**Illness:** any cough, fever, diarrhea**; Care seeking:** any care; **Type of health care:** not defined	Rural	Yes
Darmstadt 2010 [29]	Prospective	CHWs assessed neonates on days 2, 5, and 8	**Population based with minimal bias:** Live births in study population	**Illness:** 16 neonatal danger signs; **Care seeking:** any care; **Type of health care:** any treatment versus doctor, nurse, welfare visitor, medical assistant, satellite clinic, family welfare center, health complex, doctor's chamber, clinic, or hospital	Rural	Yes
Tripathy, 2010 [25]	Prospective	6 wk after delivery	**Population based with minimal bias:** Live births in study population	**Illness:** cough, fever, and/or diarrhea; **Care seeking:** care from qualified provider; **Type of health care:** qualified provide**r**	Rural	Yes
**Facility-based studies**						
**South Asia**						
Awasthi, 2006 [66]	Retrospective	Caregivers and CHWs who cared for a seriously ill neonate in past year and/or experienced a neonatal death or near death in past 2 y	**Facility-based with minimal bias:** Caregivers and CHWs who had given primary care to a newborn within six mo preceding survey	**Illness:** one or more IMNCI danger sign; **Care seeking:** any care; **Type of health care:** local medical doctor (registered or nonregistered) and traditional healer	Rural	Yes
Awasthi, 2008 [67]	Retrospective	6 wk±15 d after recruitment	**Facility-based with minimal bias:** Neonates delivered at a health center	**Illness:** one or more IMNCI danger sign; **Care seeking:** any care, including home remedies; **Type of health care:** home remedies, chemist, TH, qualified physician	Urban	Yes
Awasthi, 2009 [23]	Prospective	6–8 wk after recruitment	**Facility-based with minimal bias:** Neonates delivered at a health center	**Illness:** health related problem reported by caregiver; **Care seeking:** care from GP or NGC; **Type of health care:** GP, NGC, or NGD	Urban	Yes
**Africa region**						
Ogunlesi, 2011 [68]	Prospective	No recall period; neonates assessed at time of admission	**Facility-based with moderate bias:** Neonates with jaundice	**Illness:** jaundice; **Care seeking:** any care; **Type of health care:** private clinic, primary health center, general hospital, home care	Urban	Yes

Abbreviations: CHW, community health worker; GP, government provider; IMNCI, integrated management of childhood and neonatal illnesses, NGC, nongovernmental consultants; NGD, nongovernmental dispensers; N/A, not available; PHC, primary health care; SHC, secondary health care; TH, traditional healer.

### Type of Medical Attention/Care Sought for Sick Newborns

Table 4 presents findings from studies that reported a specific type of medical care sought and further delineating care in terms of type of facility and provider, homecare, and no care. Three studies did not identify specific type of care and were not included in this table [27],[30],[31]. Six studies reported that care sought at a secondary health center ranged from 4% to 66%. Two studies reported care sought at a primary health center (12% and 83%, respectively). Two studies reported 1% and 33% of care was sought at a pharmacy. In terms of type of health provider, nine studies reported care sought from a medically trained provider ranged from 4% to 100%. Seven studies reported care sought from an unqualified provider ranged from 1% to 83%.

**Table 4:** Community-based studies that describe type of care sought for ill or suspected ill neonates.

Primary Author, Year	Neonates with Illness/Suspected Illness (*n*)	*n* Type of Care Sought (%)^a^						
		Health Care Facility			Health Care Provider		Home Care	No Care
		Secondary Health Center	Primary Health Center	Pharmacy	Medically Trained Provider	Unqualified Provider		
**Community households surveys**								
**South Asia**								
Ahmed, 2001 [55]	740	37 (4%)			89 (12%)	607 (82%)		96 (13%)
Bhandari, 2002 [56]	9	2 (22%)						
Mohan, 2008	202		24 (12%)		8 (4%)	12 (12%)		139 (69%)
Baqui, 2008 [58]	1226				498 (41%)			
Dongre, 2008 [59]	321	56 (17%)						
Dongre, 2009 [60]	27				27 (100%)	1 (1%)		
Willis, 2009 [61]	158				62 (39%)		58 (37%)	
Chowdhury, 2011 [62]	365			2 (1%)	136 (37%)	90 (25%)		137 (38%)
**Africa region**								
Bazzano, 2008 [63]	59				23 (39%)	13 (22%)		23 (39%)
Manji, 2009 [31]	N/A		N/A (83%)			N/A (1%)	N/A (16%)	
Waiswa, 2010 [64]	6	4 (66%)		2 (33%)				
**Community-based intervention trials**								
**South Asia**								
Manandhar, 2004 [26]	**C: 1320**; I: 919	**C: 131 (10%)**; I: 219 (24%)						
Kumar, 2008 [28]	**C: 296**; ENC: 319; ENCT: 227				**C: 49 (17%)**; ENC: 78 (25%); ENCT: 76 (33%)	**C: 186 (63%)**; ENC: 152 (48%); ENCT: 106 (47%)	**C: 19 (6%)**; ENC: 27 (9%); ENCT: 22 (10%)	
Dongre, 2009 [65]	**Before: 246; After: 147**	**Before: 119 (48%)**; After: 114 (78%)						
Darmstadt, 2010 [29]	C Base: 812; C End: 400; I Base: 733; I End: 1050				**C Base: 222 (27%)**; C End: 138 (35%); I Base: 225 (31%); I End: 198 (56%)			

a Percentage reported as a proportion of all neonates with an illness or suspected illness that were included in each study. Multiple responses regarding type of care sought were permitted, as described by included studies.

Abbreviations: C, control; ENC, essential newborn care; ENCT, essential newborn care plus thermostat; I, intervention; N/A, not available.

## Discussion

While neonatal deaths comprise a staggering and increasing proportion of global deaths among children under the age of five, our review identified a paucity of data regarding care-seeking behaviors for newborn illnesses in LMICs. In LMIC settings, approximately three-quarters of neonatal deaths occur in the first week of life and nearly half occur in the first 24 h, of which more than half occur at home [32]. Timing is critical to providing neonates with appropriate care at the onset of illness and delays in the decision to seek care can have significant consequences [33]. Thus prioritizing timely and adequate care seeking for illnesses in LMICs is an essential component to improving neonatal health.

**Figure 2:**
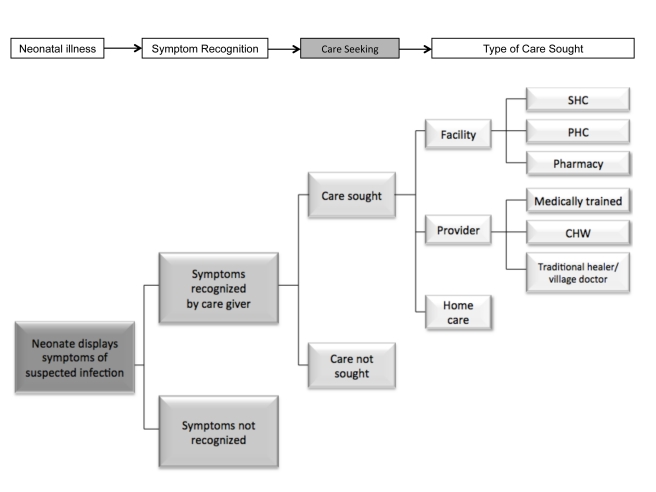
Defining care seeking conceptually. CHW, community health worker; PHC, primary health care; SHC, secondary health care.

As illustrated in Figure 2, the caregiver faces multiple decision points once the neonate displays signs of suspected illness. First, they must be able to recognize these signs; while illness recognition is fundamental in the decision to seek care, this can be particularly challenging in the neonate due to the lack of specific symptoms [4],[34],[35]. Other studies have described interventions to promote maternal recognition of neonatal illnesses [34],[36],[37]. Our review builds on this work to describe care seeking as the next step in this process of treating neonatal illnesses to reduce neonatal mortality. Understanding baseline care seeking becomes of particular importance if we were to enhance illness recognition at the home through the development of a possible point of care diagnostic test, which would then inform the caregiver as to when a neonate is ill enough to require care to be sought.

Our review found the majority of the studies occurred in rural areas in South Asia suggesting findings may not be generalizable to urban settings or other geographic regions and highlighting substantial gaps in the literature. Of the available data, the results vary greatly: studies showed anywhere from 10% to 100% of caregivers sought some form of health care for newborns with suspected illnesses, of which between 4% to 100% sought care from a trained medical provider and 4% to 66% specified receiving care at a secondary level health care facility. Despite these large variations in results, care seeking for newborn illnesses appears to be low in general and remains a key challenge to improving neonatal mortality.

Multiple factors may delay a caregiver's decision to seek care for their newborn, ranging from poor recognition of signs of illness, socio-cultural traditions regarding newborn seclusion, distance to a facility or provider, poor quality of care at facilities, lack of financial resources to access health care or transport, and the opportunity costs of missed work or childcare [35],[38],[39]. Yet there are effective strategies to address these barriers and increase the demand for newborn care. Innovative demand-side strategies have included community-based interventions to educate women in essential newborn care and birth preparedness, including illness recognition and timely, appropriate care seeking, and community mobilization initiatives to empower caregivers and communities to develop strategies to access care. Our review presents data from seven community-based intervention trials that primarily target essential newborn care, birth preparedness, and community mobilization via women's participatory groups, of which all showed an improvement of 4% to 30% in care seeking following the interventions. This finding supports similar findings from a recent meta-analysis of trials of community mobilization, where rates of institutional delivery increased by 70% in interventions groups, with a two-fold increase in studies with higher intensity mobilization activities [40]. Thus while baseline data on rates of neonatal care seeking are discouraging, there is promising evidence to suggest that innovative interventions can have a positive impact.

There is a need to simultaneously identify and strengthen supply-side strategies for neonatal health system development. However, merely increasing the availability of services, such as constructing more facilities or expanding health programs, may not directly result in an increase in the use of services. There is a role for combined strategies to increase demand for and supply of newborn health services [41]. For example, four intervention studies have explored the role of care acceptance [37],[42]–[44]. In these South Asian trials, recognition and home-management of neonatal illness by trained health care workers has lead to an increase in care acceptance and a reduction of neonatal mortality. While these studies were not included in our review as they studied care acceptance, and not care-seeking decisions, they suggest home-based neonatal care is an acceptable and feasible intervention to enhance neonatal access to appropriate health care.

While neonatal care-seeking studies need to explore the extent to which factors delay and prevent care seeking, our review recognizes the inherent complexity of constructing a neonatal care-seeking framework. Our review found that a standardized and consistent approach to care-seeking behaviors is lacking, as illustrated by the absence of universal definitions and terminology in the reviewed studies. Definitions for illness varied widely, ranging from one observed symptom to the application of multilayered algorithms. Four studies failed to provide criterion on how neonatal illness was defined, identified, or established. Care-seeking definitions varied among studies, ranging from the inclusion of any care sought, including care provided at the home, care sought from a medically trained health care provider, or at a formally established health care facility. While some studies used the term “health care providers” to include traditional healers or village doctors, others applied it only to medically qualified providers and some studies did not distinguish between these provider types. While this lack of consistency is not a new occurrence, as noted by English et al. and the WHO Multicenter Study of Severe Infection in Young Infants [4],[45], common definitions of “suspected newborn infection” and “appropriate” care-seeking behaviors have yet to be established. By addressing these inconsistencies and establishing standardized terms to identify barriers to care, future studies may be able to better generalize the factors and delays that influence neonatal care seeking.

Our findings further highlight the lack of neonatal-specific data. While published neonatal studies are mainly from South Asia, many studies have explored the care-seeking behaviors of caregivers for children under five years of age [46]–[50]. While a direct comparison of neonatal and non-neonatal care-seeking behaviors has not been studied, hospitalization rate comparisons have been published. Baqui et al., for example, found the hospitalization rate for acute lower respiratory infection in rural Bangladesh was nearly four-fold higher for children aged 1 to 5 mo than for neonates (108.9 versus 28.3 per 1,000 children-years observed) and recommended further research to identify reasons for the low hospital care usage [51]. Such findings suggest care-seeking behaviors among caregivers differ for neonates, infants, and children under five. Estimates for populations other than neonates may not be accurate or appropriate to use to approximate neonatal care-seeking patterns; this highlights the need to revise age categories used by ongoing databases and surveys, such as DHS, to specifically identify neonates and appropriately describe global and regional care-seeking behaviors.

While the study search was not limited by year, all studies that met inclusion criteria were published within the last decade, of which only four were not published within the last 5 y. Additionally, our search identified three publications that described neonatal care-seeking studies currently underway in Nepal, Malawi, and India [52]–[54]. Given the recent rise in care-seeking publications and the lack of data available before 2000, it is not possible to estimate whether care seeking for neonatal illness has improved with time. As care seeking continues to receive increasing attention within the literature, even more emphasis within the global arena is required to capture regional and time trends.

To bring about sustainable improvements in neonatal survival, changes are needed to both increase the demand for newborn care and strengthen health care systems, such as improving access and quality of care and socio-economic inequality through education and literacy initiatives. As shown in this review, there is a role for interventions within the community to encourage appropriate and timely care seeking. Community interventions provide important insight to identify and target barriers to care seeking. This paper serves as a call to action to enhance research efforts through the establishment of standardized definitions regarding illness criteria and care seeking and the classification of neonates as a specific age category in existing global databases. Additionally, community intervention research initiatives must be supported not only in South Asia, but throughout all regions of the world. By increasing global research efforts to define, understand, and address care seeking, we can continue to reduce the global burden of neonatal mortality.

## Supporting Information

Text S1 **PRISMA checklist.**
(DOC)

Text S2 **Study protocol.**
(DOCX)

## Abbreviations

LMIC: low- and middle-income country
