# Supplementary material for: Care Seeking for Neonatal Illness in Low- and Middle-Income Countries: A Systematic Review
Source: PLoS Med. 2012 Mar 6;9(3):e1001183. doi: 10.1371/journal.pmed.1001183 (PMC3295826; doi:10.1371/journal.pmed.1001183)
Supplement: Text S2 — Study protocol. (DOCX) [file pmed.1001183.s002.docx]

**Care Seeking for Neonatal Illness in Low- and Middle-Income Countries:**

**A Systematic Review**

Review Protocol

1. **Background**

Despite recent achievements to reduce child mortality, neonatal deaths continue to increase proportional to deaths among children under five, especially in low- and middle-income countries. Infections are a leading cause of neonatal death. While it is commonly thought that a lack of appropriate care seeking for ill neonates contribute to high mortality rates, estimates for care seeking behaviors in developing countries have not been studied. There is a need to systematically review the literature to determine the extent to which ill neonates seek care.

1. **Research question**

What proportion of neonates with suspected illnesses in low- and middle-income countries seek care outside the home?

1. **Search Strategy**

This combined automated and manual search will use multiple search engines and databases (see Table 1). Search terms are listed in the Appendix formatted for pubmed.

**Table 1:** Databases and Search engines

| **Database** | **Website** |
| --- | --- |
| PubMed/Medline | <http://www.ncbi.nlm.nih.gov/pubmed> |
| Embase | <http://www.embase.com/> |
| The Cochrane Library | <http://www.cochrane.org/> |
| Global Health Library | <http://www.globalhealth.org/> |
| Eldis | <http://www.eldis.org/> |
| Data Online for Population, Health, and Nutrition | <http://dolphn.aimglobalhealth.org/> |
| Reproductive Health Gateway | <http://www.k4health.org/resources/rhgateway/> |
| Basic Support for Institutionalizing Child Survival | <http://www.basics.org/> |
| Saving Newborn Lives | [http://www.savethechildren.org/site/c.8rKLIXMGIpI4E/b.6234293/k.6211/Saving_Newborn_Lives.htm#](http://www.savethechildren.org/site/c.8rKLIXMGIpI4E/b.6234293/k.6211/Saving_Newborn_Lives.htm) |
| Demographic and Health Surveys | www.statcompiler.com/ |
| Service Provision Assessments | <http://www.measuredhs.com/aboutsurveys/spa/start.cfm> |

1. **Selection Criteria**
   1. Inclusion Criteria:
      1. Studies were conducted in LMICs;
      2. Studies described care seeking for neonates with suspected illnesses; and
      3. Studies specified both a) the numbers of sick neonates, as defined by the specific study and b) the numbers of sick neonates who sought some form of health care.
   2. Exclusion Criteria:
      1. Studies did not specifically identify neonatal populations;
      2. Studies did not specify care seeking behaviors;
      3. Studies did not specify the number/proportion of neonates that sought care;
      4. Studies did not occur in LMICs; and
      5. Intervention studies that offered care in the home setting, as these studies described care acceptance behaviors (and not care seeking).
2. **Study Quality Assessment**

We will apply principles from the Cochrane Collaboration and the Working Group for Grading of Recommendations Assessment, Development and Evaluation (GRADE) to summarize the quality of the evidence for each observation as high, moderate, or low, according to study design, consistency, generalizability, precision, and reporting bias.

Studies’ methods will be examined for appropriate description and completeness. Agreement among data extractors will be evaluated if two extractors arrive at similar conclusions. If the data extractors disagree, they will discuss their position in detail, using evidence from the study in question until they reach a compromise. If they do not reach a compromise, the question at hand will be discussed with the research team during a team meeting to arrive at a compromise that the team as a whole agrees with. Specific quality variables are defined below and will be presented in a Table format (Table 2).

- 1. *Consistency* will be defined according to the selection bias of the study population. A study will be considered to have minimal selection bias if it occurred at a health facility with minimal bias and moderate selection bias if it took place at a health facility with obvious selection bias or studied a specific sub-population.
  2. *Generalizability* will be defined according to the homogeneity across studies for a given observation, highlighting if studies were predominately single center studies and/or occurred in either predominately urban or rural settings.
  3. Results will be considered *imprecise* when studies included relatively few patients, defined as a total study population was less than 300.
  4. *Reporting biased* will exist if the conclusions reached in the study were not consistent with the study’s results, conclusions were not reported, or there was a discrepancy in the reported findings.

**Table 2:** Sample Table for Quality Assessment

| Number of Studies | Study Design | | Population Representativeness | | Definition Quality | Consistency | Generalizability | Precision | | |
| --- | --- | --- | --- | --- | --- | --- | --- | --- | --- | --- |
|  | Prospective vs. retrospective | Notes | Study sample | Notes | Quality of healthcare definitions | Consistency of care seeking, care provider, and illness definitions | Generalizability to population of interest | Population with >50 | # ill neonates | # of care seeking events |
| **EXAMPLE: Care seeking associated category:** *number of studies that state ill neonates who sought care* | | | | | | | | | | |
|  |  |  |  |  |  |  |  |  |  |  |

1. **Data Extraction**

Data will be extracted through the steps outlined in Figure 1 and entered into a data extraction table (Table 3). Calculations regarding the percentage of care sought will be made by dividing the number of ill neonates that sought care by the number of ill neonates identified in the study.

**Figure 1:** Sample Flowchart for Data Extraction


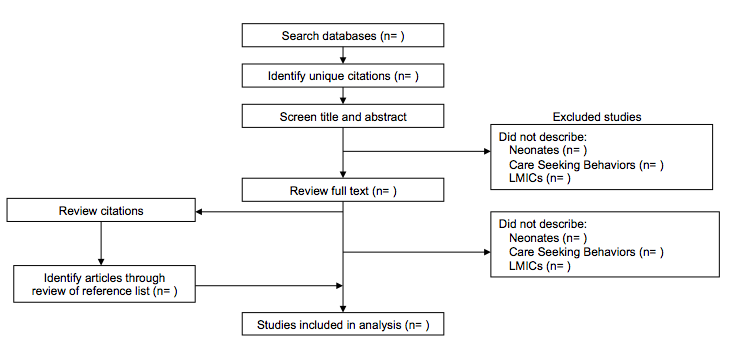


*n denotes the number of studies included in each phase of the data extraction

**Table 3:** Sample Data Extraction Table

| Author,  Year | Country | Rural/ Urban | Study design | Study Setting | Study Sample | Number of sick neonate | Number that  sought care | Percentage that sought care | Care Sought* |
| --- | --- | --- | --- | --- | --- | --- | --- | --- | --- |
|  |  |  |  |  |  |  |  |  |  |
|  |  |  |  |  |  |  |  |  |  |

*Care sought will identify where care was sought, of which example may include health care provider, health clinic, hospital, etc.

1. Synthesis

Calculations obtained from Table 3 will be tabulated and presented in a summary table (Table 4). Any threats to validity of these results will be discussed with specific reference to the quality of the studies.

**Table 4:** Sample Table of Parameter Estimate Synthesis

|  | Number of Studies | Mean | Median | Lower Bound | Upper Bound |
| --- | --- | --- | --- | --- | --- |
| Studies that describe the proportion of ill neonates that sought care |  |  |  |  |  |

1. **Study Limitations**

Currently, we do not foresee any residual validity issues inherent in our proposed search strategy. The study however may potentially be limited if the studies found in our search are not representative of global regions.

1. **Reporting**

We plan to report these findings to public health experts in child and maternal health through a planned publication in a peer-reviewed journal. This will inform further discussion regarding care seeking behaviors among ill neonates.

1. **Project timetable.**

| **Date** | **Objectives** |
| --- | --- |
| March - May | Design, test, and revise search terms |
| March - June | Run searches in database  Upload search results in Refworks**®** |
| June – July | Screen title and abstract of results |
| August - October | Obtain full texts of remaining studies and screen for inclusion/exclusion criteria |
| November - December | Analysis studies  Conduct qualitative assessment  Draft manuscript |
| January - February | Revise manuscript  Internal review |
| March | Submit for publication |

Appendix

Search terms, formatted for Pubmed®

((("infant, newborn"[MeSH Terms] OR ("infant"[All Fields] AND "newborn"[All Fields]) OR "newborn infant"[All Fields] OR "neonatal"[All Fields]) OR (neonat* OR newborn*)) AND (("Cote d'Ivoire"[All Fields] OR "eritrea"[MeSH Terms] OR ("eritrea"[MeSH Terms] OR "eritrea"[All Fields]) OR "ethiopia"[MeSH Terms] OR ("ethiopia"[MeSH Terms] OR "ethiopia"[All Fields]) OR "gambia"[MeSH Terms] OR ("gambia"[MeSH Terms] OR "gambia"[All Fields]) OR "ghana"[MeSH Terms] OR ("ghana"[MeSH Terms] OR "ghana"[All Fields]) OR "guinea"[MeSH Terms] OR ("guinea"[MeSH Terms] OR "guinea"[All Fields]) OR "guinea-bissau"[MeSH Terms] OR ("guinea-bissau"[MeSH Terms] OR "guinea-bissau"[All Fields] OR ("guinea"[All Fields] AND "bissau"[All Fields]) OR "guinea bissau"[All Fields]) OR "haiti"[MeSH Terms] OR ("haiti"[MeSH Terms] OR "haiti"[All Fields]) OR "india"[MeSH Terms] OR ("india"[MeSH Terms] OR "india"[All Fields]) OR "kenya"[MeSH Terms] OR ("kenya"[MeSH Terms] OR "kenya"[All Fields]) OR Democratic[All Fields] AND ("korea"[All Fields] OR "korea"[MeSH Terms]) OR "Kyrgyz Republic"[All Fields] OR "Lao PDR"[All Fields] OR ("lesotho"[MeSH Terms] OR "lesotho"[All Fields]) OR "liberia"[MeSH Terms] OR ("liberia"[MeSH Terms] OR "liberia"[All Fields]) OR ("madagascar"[MeSH Terms] OR "madagascar"[All Fields]) OR "malawi"[MeSH Terms] OR ("malawi"[MeSH Terms] OR "malawi"[All Fields]) OR "mali"[MeSH Terms] OR ("mali"[MeSH Terms] OR "mali"[All Fields]) OR "mauritania"[MeSH Terms] OR ("mauritania"[MeSH Terms] OR "mauritania"[All Fields]) OR "moldova"[MeSH Terms] OR ("moldova"[MeSH Terms] OR "moldova"[All Fields]) OR "mongolia"[MeSH Terms] OR ("mongolia"[MeSH Terms] OR "mongolia"[All Fields]) OR "mozambique"[MeSH Terms] OR ("mozambique"[MeSH Terms] OR "mozambique"[All Fields]) OR "myanmar"[MeSH Terms] OR ("myanmar"[MeSH Terms] OR "myanmar"[All Fields]) OR "nepal"[MeSH Terms] OR ("nepal"[MeSH Terms] OR "nepal"[All Fields]) OR "nicaragua"[MeSH Terms] OR ("nicaragua"[MeSH Terms] OR "nicaragua"[All Fields]) OR "niger"[MeSH Terms] OR ("niger"[MeSH Terms] OR "niger"[All Fields]) OR "nigeria"[MeSH Terms] OR ("nigeria"[MeSH Terms] OR "nigeria"[All Fields]) OR "North Korea"[All Fields] OR "DPRK"[All Fields] OR "pakistan"[MeSH Terms] OR ("pakistan"[MeSH Terms] OR "pakistan"[All Fields]) OR "Papua New Guinea"[All Fields] OR "rwanda"[MeSH Terms] OR ("rwanda"[MeSH Terms] OR "rwanda"[All Fields]) OR "Sao Tome and Principe"[All Fields] OR "senegal"[MeSH Terms] OR ("senegal"[MeSH Terms] OR "senegal"[All Fields]) OR "Sierra Leone"[All Fields] OR "Solomon Islands"[All Fields] OR "somalia"[MeSH Terms] OR ("somalia"[MeSH Terms] OR "somalia"[All Fields]) OR "sudan"[MeSH Terms] OR ("sudan"[MeSH Terms] OR "sudan"[All Fields]) OR "tajikistan"[MeSH Terms] OR ("tajikistan"[MeSH Terms] OR "tajikistan"[All Fields]) OR "tanzania"[MeSH Terms] OR ("tanzania"[MeSH Terms] OR "tanzania"[All Fields]) OR "east timor"[All Fields] OR "east timor"[MeSH Terms] OR ("east timor"[MeSH Terms] OR ("east"[All Fields] AND "timor"[All Fields]) OR "east timor"[All Fields] OR ("timor"[All Fields] AND "leste"[All Fields]) OR "timor leste"[All Fields]) OR "togo"[MeSH Terms] OR ("togo"[MeSH Terms] OR "togo"[All Fields]) OR "uganda"[MeSH Terms] OR ("uganda"[MeSH Terms] OR "uganda"[All Fields]) OR "uzbekistan"[MeSH Terms] OR ("uzbekistan"[MeSH Terms] OR "uzbekistan"[All Fields]) OR "vietnam"[MeSH Terms] OR ("vietnam"[MeSH Terms] OR "vietnam"[All Fields]) OR "yemen"[MeSH Terms] OR ("yemen"[MeSH Terms] OR "yemen"[All Fields]) OR "Republic of Yemen"[All Fields] OR "democratic republic of the congo"[All Fields] OR "democratic republic of the congo"[MeSH Terms] OR ("democratic republic of the congo"[MeSH Terms] OR ("democratic"[All Fields] AND "republic"[All Fields] AND "congo"[All Fields]) OR "democratic republic of the congo"[All Fields] OR "zaire"[All Fields]) OR "zambia"[MeSH Terms] OR ("zambia"[MeSH Terms] OR "zambia"[All Fields]) OR "zimbabwe"[MeSH Terms] OR ("zimbabwe"[MeSH Terms] OR "zimbabwe"[All Fields]) OR "albania"[MeSH Terms] OR ("albania"[MeSH Terms] OR "albania"[All Fields]) OR "algeria"[MeSH Terms] OR ("algeria"[MeSH Terms] OR "algeria"[All Fields]) OR "angola"[MeSH Terms] OR ("angola"[MeSH Terms] OR "angola"[All Fields]) OR "armenia"[MeSH Terms] OR ("armenia"[MeSH Terms] OR "armenia"[All Fields]) OR "azerbaijan"[MeSH Terms] OR ("azerbaijan"[MeSH Terms] OR "azerbaijan"[All Fields]) OR "byelarus"[MeSH Terms] OR ("byelarus"[MeSH Terms] OR "byelarus"[All Fields] OR "belarus"[All Fields]) OR "bolivia"[MeSH Terms] OR ("bolivia"[MeSH Terms] OR "bolivia"[All Fields]) OR "Bosnia and Herzegovina"[All Fields] OR "brazil"[MeSH Terms] OR ("brazil"[MeSH Terms] OR "brazil"[All Fields]) OR "bulgaria"[MeSH Terms] OR ("bulgaria"[MeSH Terms] OR "bulgaria"[All Fields]) OR "Cape Verde"[All Fields] OR "china"[MeSH Terms] OR ("china"[MeSH Terms] OR "china"[All Fields]) OR "colombia"[MeSH Terms] OR ("colombia"[MeSH Terms] OR "colombia"[All Fields]) OR "cuba"[MeSH Terms] OR ("cuba"[MeSH Terms] OR "cuba"[All Fields]) OR "djibouti"[MeSH Terms] OR ("djibouti"[MeSH Terms] OR "djibouti"[All Fields]) OR "Dominican Republic"[All Fields] OR "ecuador"[MeSH Terms] OR ("ecuador"[MeSH Terms] OR "ecuador"[All Fields]) OR "egypt"[MeSH Terms] OR ("egypt"[MeSH Terms] OR "egypt"[All Fields]) OR "Arab Republic of Egypt"[All Fields] OR "El Salvador"[All Fields] OR "fiji"[MeSH Terms] OR ("fiji"[MeSH Terms] OR "fiji"[All Fields]) OR "georgia republic"[MeSH Terms] OR "georgia"[tiab] OR "georgia (republic)"[MeSH Terms] OR "guatemala"[MeSH Terms] OR ("guatemala"[MeSH Terms] OR "guatemala"[All Fields]) OR "guyana"[MeSH Terms] OR ("guyana"[MeSH Terms] OR "guyana"[All Fields]) OR "honduras"[MeSH Terms] OR ("honduras"[MeSH Terms] OR "honduras"[All Fields]) OR "indonesia"[MeSH Terms] OR ("indonesia"[MeSH Terms] OR "indonesia"[All Fields]) OR "iran"[MeSH Terms] OR ("iran"[MeSH Terms] OR "iran"[All Fields]) OR "Islamic Republic of Iran"[All Fields] OR "iraq"[MeSH Terms] OR ("iraq"[MeSH Terms] OR "iraq"[All Fields]) OR "jamaica"[MeSH Terms] OR ("jamaica"[MeSH Terms] OR "jamaica"[All Fields]) OR "jordan"[MeSH Terms] OR ("jordan"[MeSH Terms] OR "jordan"[All Fields]) OR "kazakhstan"[MeSH Terms] OR ("kazakhstan"[MeSH Terms] OR "kazakhstan"[All Fields]) OR "micronesia"[All Fields] OR "micronesia"[MeSH Terms] OR ("micronesia"[MeSH Terms] OR "micronesia"[All Fields] OR "kiribati"[All Fields]) OR "macedonia republic"[MeSH Terms] OR ("macedonia (republic)"[MeSH Terms] OR ("macedonia"[All Fields] AND "(republic)"[All Fields]) OR "macedonia (republic)"[All Fields] OR "macedonia"[All Fields]) OR "FYR of Macedonia"[All Fields] OR "Former Yugoslav Republic of Macedonia"[All Fields] OR "indian ocean islands"[All Fields] OR "indian ocean islands"[MeSH Terms] OR ("indian ocean islands"[MeSH Terms] OR ("indian"[All Fields] AND "ocean"[All Fields] AND "islands"[All Fields]) OR "indian ocean islands"[All Fields] OR "maldives"[All Fields]) OR "Marshall Islands"[All Fields] OR "micronesia"[MeSH Terms] OR ("micronesia"[MeSH Terms] OR "micronesia"[All Fields]) OR "Federated States of Micronesia"[All Fields] OR "morocco"[MeSH Terms] OR ("morocco"[MeSH Terms] OR "morocco"[All Fields]) OR "namibia"[MeSH Terms] OR ("namibia"[MeSH Terms] OR "namibia"[All Fields]) OR "paraguay"[MeSH Terms] OR ("paraguay"[MeSH Terms] OR "paraguay"[All Fields]) OR "peru"[MeSH Terms] OR ("peru"[MeSH Terms] OR "peru"[All Fields]) OR "philippines"[MeSH Terms] OR ("philippines"[MeSH Terms] OR "philippines"[All Fields]) OR "romania"[MeSH Terms] OR ("romania"[MeSH Terms] OR "romania"[All Fields]) OR "samoa"[MeSH Terms] OR ("samoa"[MeSH Terms] OR "samoa"[All Fields]) OR "Serbia and Montenegro"[All Fields] OR "Sri Lanka"[All Fields] OR "suriname"[MeSH Terms] OR ("suriname"[MeSH Terms] OR "suriname"[All Fields]) OR "swaziland"[MeSH Terms] OR ("swaziland"[MeSH Terms] OR "swaziland"[All Fields]) OR "Syrian Arab Republic"[All Fields] OR "syria"[MeSH Terms] OR ("syria"[MeSH Terms] OR "syria"[All Fields]) OR "thailand"[MeSH Terms] OR ("thailand"[MeSH Terms] OR "thailand"[All Fields]) OR "tonga"[MeSH Terms] OR ("tonga"[MeSH Terms] OR "tonga"[All Fields]) OR "tunisia"[MeSH Terms] OR ("tunisia"[MeSH Terms] OR "tunisia"[All Fields]) OR "turkmenistan"[MeSH Terms] OR ("turkmenistan"[MeSH Terms] OR "turkmenistan"[All Fields]) OR "ukraine"[MeSH Terms] OR ("ukraine"[MeSH Terms] OR "ukraine"[All Fields]) OR "vanuatu"[MeSH Terms] OR ("vanuatu"[MeSH Terms] OR "vanuatu"[All Fields]) OR "West Bank and Gaza"[All Fields] OR "American Samoa"[All Fields] OR "Antigua and Barbuda"[All Fields] OR "argentina"[MeSH Terms] OR ("argentina"[MeSH Terms] OR "argentina"[All Fields]) OR "barbados"[MeSH Terms] OR ("barbados"[MeSH Terms] OR "barbados"[All Fields]) OR "belize"[MeSH Terms] OR ("belize"[MeSH Terms] OR "belize"[All Fields]) OR "botswana"[MeSH Terms] OR ("botswana"[MeSH Terms] OR "botswana"[All Fields]) OR "chile"[MeSH Terms] OR ("chile"[MeSH Terms] OR "chile"[All Fields]) OR "Costa Rica"[All Fields] OR "croatia"[MeSH Terms] OR ("croatia"[MeSH Terms] OR "croatia"[All Fields]) OR "Czech Republic"[All Fields] OR "dominica"[MeSH Terms] OR ("dominica"[MeSH Terms] OR "dominica"[All Fields]) OR "Equatorial Guinea"[All Fields] OR "estonia"[MeSH Terms] OR ("estonia"[MeSH Terms] OR "estonia"[All Fields]) OR "gabon"[MeSH Terms] OR ("gabon"[MeSH Terms] OR "gabon"[All Fields]) OR "grenada"[MeSH Terms] OR ("grenada"[MeSH Terms] OR "grenada"[All Fields]) OR "hungary"[MeSH Terms] OR ("hungary"[MeSH Terms] OR "hungary"[All Fields]) OR "latvia"[MeSH Terms] OR ("latvia"[MeSH Terms] OR "latvia"[All Fields]) OR "lebanon"[MeSH Terms] OR ("lebanon"[MeSH Terms] OR "lebanon"[All Fields]) OR "libya"[MeSH Terms] OR ("libya"[MeSH Terms] OR "libya"[All Fields]) OR "lithuania"[MeSH Terms] OR ("lithuania"[MeSH Terms] OR "lithuania"[All Fields]) OR "malaysia"[MeSH Terms] OR ("malaysia"[MeSH Terms] OR "malaysia"[All Fields]) OR "mauritius"[MeSH Terms] OR ("mauritius"[MeSH Terms] OR "mauritius"[All Fields]) OR "comoros"[All Fields] OR "comoros"[MeSH Terms] OR ("comoros"[MeSH Terms] OR "comoros"[All Fields] OR "mayotte"[All Fields]) OR "mexico"[MeSH Terms] OR ("mexico"[MeSH Terms] OR "mexico"[All Fields]) OR "Northern Mariana Islands"[All Fields] OR "oman"[MeSH Terms] OR ("oman"[MeSH Terms] OR "oman"[All Fields]) OR "palau"[MeSH Terms] OR ("palau"[MeSH Terms] OR "palau"[All Fields]) OR "panama"[MeSH Terms] OR ("panama"[MeSH Terms] OR "panama"[All Fields]) OR "poland"[MeSH Terms] OR ("poland"[MeSH Terms] OR "poland"[All Fields]) OR "Russian Federation"[All Fields] OR "seychelles"[MeSH Terms] OR ("seychelles"[MeSH Terms] OR "seychelles"[All Fields]) OR "Slovak Republic"[All Fields] OR "South Africa"[All Fields] OR "St. Kitts and Nevis"[All Fields] OR "St. Lucia"[All Fields] OR "St. Vincent and the Grenadines"[All Fields] OR "Trinidad and Tobago"[All Fields] OR "turkey"[MeSH Terms] OR ("turkey"[MeSH Terms] OR "turkey"[All Fields]) OR "uruguay"[MeSH Terms] OR ("uruguay"[MeSH Terms] OR "uruguay"[All Fields]) OR "venezuela"[MeSH Terms] OR ("venezuela"[MeSH Terms] OR "venezuela"[All Fields]) OR "developing countries"[All Fields] OR "less developed countries"[All Fields] OR "third-world countries"[All Fields] OR "under-developed countries"[All Fields] OR "poOR countries"[All Fields] OR "less developed countries"[All Fields] OR "under developed countries"[All Fields] OR "less developed nations"[All Fields] OR "third world nations"[All Fields] OR "under developed nations"[All Fields] OR "developing nations"[All Fields] OR "poOR nations"[All Fields] OR "poor economies"[All Fields] OR "developing economies"[All Fields] OR "less developed economies"[All Fields] OR "myanmar"[MeSH Terms] OR ("myanmar"[MeSH Terms] OR "myanmar"[All Fields] OR "burma"[All Fields]) OR "Czechoslovakia"[All Fields] OR "Democratic Republic of Congo"[All Fields] OR "French Guiana"[All Fields] OR "East Timor"[All Fields] OR "laos"[MeSH Terms] OR ("laos"[MeSH Terms] OR "laos"[All Fields]) OR "North Korea"[All Fields] OR "Ivory Coast"[All Fields] OR "Republic of Georgia"[All Fields] OR "Republic of Yemen"[All Fields] OR "Republic of Zaire"[All Fields] OR "slovakia"[MeSH Terms] OR ("slovakia"[MeSH Terms] OR "slovakia"[All Fields]) OR "Soviet Union"[All Fields] OR "suriname"[MeSH Terms] OR ("suriname"[MeSH Terms] OR "suriname"[All Fields] OR "surinam"[All Fields]) OR "ussr"[MeSH Terms] OR ("ussr"[MeSH Terms] OR "ussr"[All Fields]) OR "samoa"[MeSH Terms] OR ("samoa"[MeSH Terms] OR "samoa"[All Fields]) OR "yugoslavia"[MeSH Terms] OR ("yugoslavia"[MeSH Terms] OR "yugoslavia"[All Fields]) OR "democratic republic of the congo"[MeSH Terms] OR ("democratic republic of the congo"[MeSH Terms] OR ("democratic"[All Fields] AND "republic"[All Fields] AND "congo"[All Fields]) OR "democratic republic of the congo"[All Fields] OR "zaire"[All Fields]) OR "asia"[MeSH Terms] OR ("asia"[MeSH Terms] OR "asia"[All Fields]) OR "West Indies"[All Fields] OR "polynesia"[MeSH Terms] OR ("polynesia"[MeSH Terms] OR "polynesia"[All Fields]) OR "micronesia"[MeSH Terms] OR ("micronesia"[MeSH Terms] OR "micronesia"[All Fields]) OR "Middle East"[All Fields] OR "africa"[MeSH Terms] OR ("africa"[MeSH Terms] OR "africa"[All Fields]) OR "Latin America"[All Fields] OR "Central America"[All Fields] OR "South America"[All Fields] OR ("west indies"[MeSH Terms] OR ("west"[All Fields] AND "indies"[All Fields]) OR "west indies"[All Fields]) OR "west indies"[MeSH Terms] OR "caribbean region"[MeSH Terms] OR ("west indies"[MeSH Terms] OR ("west"[All Fields] AND "indies"[All Fields]) OR "west indies"[All Fields] OR "caribbean"[All Fields] OR "caribbean region"[MeSH Terms] OR ("caribbean"[All Fields] AND "region"[All Fields]) OR "caribbean region"[All Fields]) OR "caribbean region"[MeSH Terms] OR Hispanola[All Fields] OR "Southeast Asia"[All Fields] OR "Sub-Saharan Africa"[All Fields] OR "Eastern Europe"[All Fields] OR Balkans[All Fields]) OR "Developing Countries"[MeSH Terms] OR "Developing Country"[All Fields] OR "Developing Countries"[All Fields] OR "Low Resource Setting"[All Fields] OR "Low Resource Settings"[All Fields] OR Bangladesh[mesh] OR bangladesh[all fields])) AND ("care seeking"[All Fields] OR "care-seeking"[All Fields] OR "careseeking"[All Fields] OR "birth preparedness"[All Fields])
